# Lipid profiles in schizophrenia associated with clinical traits: a five year follow-up study

**DOI:** 10.1186/s12888-016-1006-3

**Published:** 2016-08-25

**Authors:** Dag K. Solberg, Håvard Bentsen, Helge Refsum, Ole A. Andreassen

**Affiliations:** 1 Institute for Military Psychiatry, Norwegian Defense Medical Services, Pb 1550 Sentrum, 0015 Oslo, Norway; 2 Center for Psychopharmacology, Diakonhjemmet Hospital, Oslo, Norway; 3 NORMENT, KG Jebsen Centre for Psychosis Research, Institute of Clinical Medicine, University of Oslo, Oslo, Norway; 4 Division of Mental Health and Addiction, Oslo University Hospital, Oslo, Norway

## Abstract

**Background:**

Alterations in serum and membrane lipids may be involved in schizophrenia pathophysiology. It is not known whether lipid profiles are associated with disease severity or current symptom level.

**Methods:**

Clinical and lipid data were gathered from 55 patients with schizophrenia admitted to psychiatric emergency wards in an acute stage of the disease (T_1_). The patients were re-examined after 5 years at a stable phase (T_2_). The clinical assessments included Positive and Negative Syndrome Scale (PANSS total, positive, negative) and Global Assessment of Functioning (GAF S, symptom and F, function). Serum lipids (cholesterol and triglyceride) and membrane polyunsaturated fatty acids (PUFA, LCPUFA) were measured. Healthy controls were recruited among hospital workers.

**Results:**

Serum triglyceride was significantly higher in patients with schizophrenia compared to healthy controls both at T_1_ and T_2_ (*p <* 0.001), while serum cholesterol did not differ significantly. The levels of serum lipids in patients remained stable over time. At T_1_, serum lipids and symptoms were not significantly correlated. At T_2_, higher serum lipids were associated with more severe symptoms and poorer functioning. Higher serum lipid levels at T_1_ were associated with more severe symptoms and poorer functioning at T_2_; cholesterol with GAF-S (*p <* 0.05), triglyceride with PANSS total (*p <* 0.05), GAF-S (*p <* 0.01) and GAF-F (*p <* 0.01). Membrane lipids were significantly lower in the patient group compared to healthy controls at T_1_ (PUFA *p <* 0.001, LCPUFA *p <* 0.001), but not at T_2_. Membrane lipids were not significantly correlated with symptoms at T_1_, but significantly associated with negative symptoms and functioning at T_2_ as previously reported.

**Conclusions:**

The present findings suggest different roles of membrane and serum lipids in schizophrenia pathophysiology. To further elucidate the relation of lipid biology to disease traits, replication in independent studies of longitudinal samples are warranted.

## Background

Abnormal lipid biology may play a significant role in the pathophysiology of schizophrenia. Most studies show that patients with schizophrenia have higher levels of serum lipids (cholesterol and triglyceride) than a healthy population [1, 2]. This dyslipidemia has been regarded as a result of antipsychotic medication and lifestyle factors [3], but dyslipidemia has also been demonstrated in unmedicated schizophrenia patients [4–7]. Altered metabolism of membrane lipids (polyunsaturated fatty acids, PUFA) is another aspect of lipid biology suggested to be involved in schizophrenia pathophysiology [8]. Lower levels of PUFA in cell membranes have been found in schizophrenia [9, 10], both in the acute and chronic stage of the disease [11].

The course and outcome of schizophrenia is regarded as heterogeneous. The nature of the relation between lipid profiles, lipid metabolism and clinical characteristics of schizophrenia is mainly unknown. Particularly, it is not known whether lipid profiles are associated with the disease itself, and / or current symptoms. Conflicting findings of associations between lipid levels and symptom severity may represent fluctuations of lipid levels as the disease progresses [12, 13]. It is possible that lipid levels are stable while symptoms, especially positive psychotic symptoms, fluctuate during the course of the disease. The presence of abnormal lipid metabolism from the onset of the disease that remains stable independent of disease symptoms and antipsychotic treatment, may suggest that lipid abnormalities are a disease trait, and thus involved in the pathophysiological development of the disease, as suggested for cholesterol [14]. Lipids that are aberrant during an acute psychotic episode of schizophrenia and normalized after the acute episode may indicate a role for lipids in relation to disease symptoms, which has been suggested for membrane lipids (PUFA) [15]. The role of lipid biology in relation to disease symptoms can best be investigated in a longitudinal study, following patients during different stages of the disease.

Several lines of evidence suggest that the pathophysiology of schizophrenia involves immune- and inflammatory pathways, integrated with redox-regulation [16, 17]. It has been suggested that the composition of membrane lipids is abnormal [18, 19], potentially due to disturbed redox-regulation [20, 21]. Oxidative stress can also affect serum lipids and cause dyslipidemia [22–24]. In schizophrenia, levels of both serum- and membrane lipids seem aberrant [9, 25, 26] Thus, an alteration in redox-regulation can be a common factor linking abnormalities of both serum and membrane lipids in schizophrenia. A change in membrane lipid composition in neuronal cells can affect neurotransmission, symptoms and behavior in schizophrenia [27]. Both serum and membrane lipids have been found to predict the outcome of treatment [28, 29]. We hypothesize that both serum lipid and membrane lipid alterations may be involved in the pathophysiology of schizophrenia.

We have reported earlier that symptom levels were positively associated with two types of lipids, both serum lipids and membrane lipids in patients with schizophrenia [26]. How these relationships change over time is unknown. Here we investigate with a longitudinal design, using a sample with repeated assessment, if the lipid profiles vary in relation to clinical characteristics; positive and negative symptoms (PANSS) and general symptoms and functioning (GAF). We hypothesize that there is a core abnormality in both membrane and serum lipid systems in schizophrenia, reflected in abnormal membrane and serum lipid levels, and this is independent of disease phase. In order to test this hypothesis, we examined a group of patients and healthy controls at baseline and after 5 years follow-up.

The aims of the current study are first to determine if there are differences in lipid profiles (serum lipids and membrane lipids) between people with schizophrenia and healthy controls and if they are stable over a 5 years period. The second aim is to explore the relationship between lipid profiles and clinical characteristics during the course of schizophrenia. We measured the levels of serum and membrane lipids at admission to emergency psychiatric wards and after 5 years follow-up in out-patients clinics or at long-term care facilities, and their association with the disease and clinical symptoms. Repeated measures of lipid levels during the course of schizophrenia and their relationship with clinical symptoms may elucidate whether lipid profiles are associated with stable disease characteristics (traits).

## Methods

### Participants

In a longitudinal study, socioeconomic, clinical and biological data were gathered from a group of 55 patients with schizophrenia and schizoaffective disorders. The patients were a sub-sample of a group of 99 patients participating in a trial of an omega-3 fatty acid and antioxidants in schizophrenia [30]. The patients were recruited when admitted to psychiatric emergency wards in southern Norway in 2001 to 2003 (T_1_) [30]. They were examined again between 2006 and 2010 (T_2_), with a mean follow-up time of 61 months. Of the 44 patients not included in the follow-up study, 21 did not wish to participate, twelve were not located, nine were dead, and two had moved to other regions of Norway. The sub-sample of 55 patients did not statistically differ from the main sample of 99 patients with regard to demographics or main outcome variables at T_1_. When examined at the inclusion (T_1_), the patients were admitted to an emergency psychiatric ward and thus considered in an acute stage of the disease. Five years later (T_2_), the patients were treated at out-patient clinics or at psychiatric long term care facilities, and considered to be in a more stable chronic stage. The patients were screened for somatic illness at inclusion and at follow-up. The data from an overlapping sample at T_2_ have been presented earlier [26]. In addition, healthy controls were recruited among hospital employees from the same age group as patients. At T_1_, 20 healthy controls were included. At T_2,_ 51 healthy controls were examined. Of these, 16 were a subsample from T_1_, and an additional 35 healthy participants were included as healthy controls at T_2_. To be included as healthy controls, participants and their first-degree relatives could not have any ongoing or past severe psychiatric disorder. This was determined with an interview assessing severe mental illness, and screening for ongoing and past psychiatric disorder using the Mini International Neuropsychiatric Interview (MINI). In addition, their physical health was assessed with self-report and short screening interview addressing current or history of somatic illness in the healthy control participants.

Demographics of patients and healthy controls are reported in Table 1. The study was approved by the Regional Committee for Medical Research Ethics. All participants gave written informed consent. Inclusion of patients ended in June of 2010 and results were submitted in 2015. This delay was partly caused by technological difficulties which led to re-analyzes and data analysis could not start until late 2011, and partly due to clinical duties for the first author.

**Table 1:** Demographics

	Patients T_1_	Healthy controls T_1_	Patients T_2_	Healthy controls T_2_
	*n =* 55	*n =* 20	*n =* 55	*n =* 51¤
Age	26.5 ± 6.1	31.1 ± 5.3	31.3 ± 5.7	33.0 ± 6.1
Sex (% male)	69.1	55.0	69.1	54.9
Smokers (%)	31 (56.4)	7 (35.0)	29 (52.7)	9 (17.6)
Primary school (%) ^a^	14 (25.5)	3 (15.0)	9 (17.0)	1 (2.0)
Secondary school (%)	30 (54.5)	8 (40.0)	30 (56.6)	8 (16.0)
University / college (%)	11 (20.0)	9 (45.0)	14 (26.4)	41 (82.0)
PANSS total	81 (71,93)		82 (57,101)	
PANSS negative	22 (16,27)		25 (18,31)	
PANSS positive	17 (13,21)		16 (11,19)	
GAF-S	35 (30,40)		45 (37,58)	
GAF-F	37 (31,40)		47 (38,58)	

*PANSS* positive and negative symptoms scale

*PANSS total* positive and negative symptoms scale

*PANSS positive* positive component: items P1 + P3 + P5 + P6 + G9

*PANSS negative* negative component: items N1 + N2 + N3 + N4 + N6 + G7 + G8 + G16

*GAF* global assessment of functioning, *S* symptoms, *F* functioning

*Age* mean ± standard deviation

PANSS, GAF: median (25,75 percentiles)

^a^ data missing from patient T_2_ *n =* 2, control T_2_ = 1

¤ 16 healthy controls from T_1_, 35 additional healthy controls included at T_2_

### Clinical assessment

All patients were diagnosed with the Structural Clinical Interview for DSM-IV (SCID). To measure the severity of symptoms the Positive and Negative Syndrome Scale Structured Interview Version (SCI-PANSS), and Global Assessment of Functioning (GAF) were used. In addition to PANSS total, we reported positive and negative PANSS components from a model established by van der Gaag et al. [31]. The split version of GAF was used, which includes Symptom (GAF-S) and Functioning (GAF-F) scales [32]. At T_1_ the patients were assessed by a group of 16 investigators from the participating hospitals. DKS was among the investigators at T_1_, who underwent trainings sessions and the interrater reliability was assessed by rating ten (median) videotaped interviews [33]. At T_2_, all patients were assessed by the same clinical investigator (DKS).

### Biochemical assays

Blood for lipid analyses was sampled after overnight fasting. Serum lipids and membrane lipids were measured. At T_1_ serum lipids were not obtained from healthy controls.

Serum lipids (cholesterol and triglyceride) were analyzed at the Department of Clinical Chemistry at Aker Hospital (T_1_) and Diakonhjemmet Hospital (T_2_) with standard enzymatic methods from Roche Diagnostics Norge AS, Oslo, Norway. For analyses of polyunsaturated fatty acids washed blood cells were stored at−70 °C and sent within 3 months in dry ice to Mylnefield Research Services LTD, Dundee, United Kingdom, who did the analysis. The lipids were extracted, converted into fatty acid methyl esters, and analyzed by gas chromatography, yielding fatty acid profiles. In total 28 species of fatty acids from C14:0 to C24:1 is reported as micrograms per gram of RBC (red blood cells). The sum of omega-3 fatty acids is C18:3 + C18:4 + C20:3 + C20:5 + C22:5 + C22:6. The sum of omega-6 fatty acids is C18:2 + C18:3 + C20:2 + C20:3 + C20:4 + C22:4 + C22:5. The sum of omega-3 and omega-6 is named polyunsaturated fatty acids (PUFA). The sum of PUFA with 20 or 22 carbon atoms is named long-chain polyunsaturated fatty acids (LCPUFA).

### Pharmacological treatment

Use of antipsychotic medication may affect the levels of serum lipids. The current and previous use of antipsychotics and other pharmacological agents in the patients were registered from interviews and medical records information. At T_2_, detectable levels of antipsychotic drug in serum samples were measured to control for adherence (therapeutic drug monitoring). Use of medication for dyslipidemia was registered.

### Statistics

All statistical analyses were performed using SPSS version 20 (SPSS Inc, Chicago, IL, USA / IBM, New York, USA). Demographical and clinical variables are presented as average values or proportions. Parametric or non-parametric tests were chosen depending on the distribution of variables. The Wilcoxon signed rank test was used to compare lipid levels within patients. The Mann–Whitney test was used to compare lipid levels between patients and healthy controls. Two-sided tests were used, and the significance level was set to *p <* 0.05. Spearman’s correlation coefficients (r_s_) were used to evaluate the relationship between lipid data and clinical symptoms (PANSS and GAF).

## Results

### Clinical characteristics

The patients had significantly lower GAF score at T_1_ than T_2_; GAF-S (*p <* 0.001) and GAF-F (*p <* 0.001), while the PANSS scores were not significantly different between T_1_ and T_2_. See Table 1 for details.

### Use of medication

No patients or healthy controls used medication for dyslipidemia. At T_1_ (admitted to an emergency psychiatric ward) all 55 of the patients used antipsychotic medication, but only 26 (47.3 %) used antipsychotic medication before admission. At T_2_, 44 patients (80 %) used antipsychotic medication.

### Repeated lipid measures

#### Serum lipids

The levels of triglyceride were significantly higher in patients with schizophrenia than in healthy controls both at T_1_ (*p <* 0.001), and at T_2_ (*P <* 0.001) (Table 2). The difference in cholesterol between patients (T_1_ and T_2_) and healthy controls (T_2_) was not significant. Fasting serum lipid data were not available in the healthy control group at T_1_. The levels of serum lipids (triglyceride and cholesterol) remained stable in the patient group over time, with no significant difference between T_1_ and T_2_. In the patient group, serum lipid levels remained stable over the 5 year follow-up period, illustrated with significantly correlated levels at T_1_ and T_2_ for both cholesterol (*r*_*s*_ = 0.63, *p <* 0.001) and triglyceride (*r*_*s*_ = 0.54, *p <* 0.001).

**Table 2:** Serum and membrane lipids, patients and healthy controls, case–control and longitudinal data

	Patients T_1_	Patients T_2_	Healthy controls T_1_	Healthy controls T_2_
	*n =* 55	*n =* 55	*n =* 20	*n =* 51
PUFA	434 (171,507)	471 (440,513)^***^	478 (446,495)^***^	470.0 (437,508)
LCPUFA	283 (89,337)	308 (291,334)^***^	307 (293,330)^***^	306 (287,336)
S-cholesterol	5.00 ± 1.12	5.36 ± 1.20	¤	5.05 ± 0.84
S-triglyceride	1.50 (0.80,2.48)^###^	1.33 (0.95,2.66)^###^	¤	0.85 (0.59,1.12)

^***^ *P <*0.001 vs. patients T_1_, ^###^ *P <* 0.001 vs. healthy controls T_2_

¤not measured

Serum cholesterol: normally distributed, mean ± standard deviation

Serum triglyceride, PUFA, LCPUFA: non-normally distributed, median (25,75 percentiles)

Patients T_1_ vs patients T_2_: Wilcoxon signed rank test

Patients T_1_ and T_2_ vs healthy controls T_1_: Mann–Whitney test

Healthy control T_1_ vs healthy controls T_2_: Wilcoxon signed rank test

PUFA = omega-3 + omega-6 polyunsaturated fatty acids in red blood cells

LCPUFA = omega-3 + omega-6 PUFA with 20 or more carbon atoms in red blood cells

#### Membrane lipids

Membrane lipids were significantly lower in the patient group compared to healthy controls at T_1_, both for PUFA (*p <* 0.001) and LCPUFA (*P <* 0.001) (Table 2). At T_2_, there was no difference in the levels of membrane lipids between patients and healthy controls. The patients with schizophrenia had significantly lower levels of membrane lipids at T_1_ than at T_2_, both for PUFA (*p <* 0.001) and LCPUFA (*p <* 0.001) (Table 2). As reported earlier, the distribution of PUFA was bimodal at T_1,_ defining a low and a high PUFA group, and normal at T_2_ [9]. The mean levels of membrane lipids increased only from T_1_ to T_2_ in patients belonging to the low PUFA group at T_1_. PUFA levels at T_1_ and T_2_ were not significantly associated. For healthy controls, there was no significant difference between membrane lipid levels at T_1_ and T_2_.

### Relationship between lipid levels and clinical characteristics

#### Serum lipids

In general, the associations between serum lipids and symptoms and functioning were stronger at T_2_ than T_1_ (Table 3). There were no significant relationship between the lipid levels and clinical variables at T_1_. As reported earlier, there was a significant correlation between cholesterol and GAF-S at T_2_ (*p =* 0.02), and significant correlations between triglyceride and GAF-S (*p =* 0.001), GAF-F (*p =* 0.01) and PANSS positive (*p =* 0.04). The associations at T_2_ indicate that higher serum lipids were related to poorer functioning and more severe symptoms.

**Table 3:** Association (r_s_) between lipid levels in serum and cell membranes and clinical characteristics in patients

	S-triglyceride		S-cholesterol		RBC PUFA		RBC LCPUFA	
	T_1_	T_2_	T_1_	T_2_	T_1_	T_2_	T_1_	T_2_
PANSS total	0.06	0.26	0.01	0.15	0.08	0.14	0.09	0.31*
PANSS negative	0.18	0.26	0.19	0.11	0.21	0.32*	0.20	0.52***
PANSS positive	−0.04	0.28*	−0.03	0.22	−0.02	0.01	−0.07	0.11
GAF-S	0.13	−0.48***	0.26	−0.30*	−0.24	−0.22	−0.26	−0.32*
GAF-F	0.07	−0.32*	0.24	−0.23	−0.19	−0.21	−0.23	−0.29*

*P ≤ 0.05, **P ≤ 0.01, ***P ≤ 0.001

Spearman’s correlation coefficient is reported

*PANSS* positive and negative syndrome scale

*GAF* global assessment of functioning, *S* symptoms, *F* functioning)

*PANSS Positive* positive component; items P1 + P3 + P5 + P6 + G9

*PANSS Negativ*e negative component; items N1 + N2 + N3 + N4 + N6 + G7 + G8 + G16

PUFA = omega-3 + omega- 6 polyunsaturated fatty acids in red blood cells (RBC)

LCPUFA = omega-3 + omega-6 PUFA with 20 or more carbon atoms in red blood cells (RBC)

We also investigated associations between serum lipid levels at T_1_, and symptom levels at T_2_. The results are reported in Table 4. Triglyceride at T_1_ was significantly correlated to PANSS total (*p =* 0.04), GAF-S (*p =* 0.003) and GAF-F (*p =* 0.006) at T_2_. For cholesterol at T_1_ there was significant correlation to GAF-S at T_2_ (*p =* 0.05). All these associations indicate that higher serum lipids at T_1_ were related to poorer functioning and more severe symptoms at T_2_.

**Table 4:** Association (r_s_) between serum lipid levels in serum at T_1_ and clinical characteristics at T_2_

	S-triglyceride T_1_	S-cholesterol T_1_
PANSS positive T_2_	0.21	0.06
PANSS negative T_2_	0.27	0.15
PANSS total T_2_	0.28*	0.11
GAF-S T_2_	−0.41**	−0.28*
GAF-F T_2_	−0.39**	−0.26

*0.01 ≤ *P <* 0.05, **P < 0.01

Spearman’s correlation coefficient is reported

There was no significant association between levels of *PUFA* (polyunsaturated fatty acids) / *LCPUFA* (long chain polyunsaturated fatty acids) at T_1_ and symptoms at T_2_

*PANSS* positive and negative syndrome scale

*GAF* global assessment of functioning, *S* symptoms, *F* functioning)

*PANSS positive* items P1 + P3 + P5 + P6 + G9

*PANSS negative* items N1 + N2 + N3 + N4 + N6 + G7 + G8 + G16

#### Membrane lipids

As for serum lipids, the associations between membrane lipids and symptoms and functioning were stronger at T_2_ than T_1_ (Table 3). There were no significant relationships between these lipids and clinical variables at T_1_. At T_2,_ there were significant correlations between LCPUFA and PANSS negative (*p =* 0.001), PANSS total (*p =* 0.02), GAF-S (*p =* 0.02) and GAF-F (*p =* 0.04). PUFA at T_2_ was correlated to PANSS negative (*p =* 0.02). All associations at T_2_ indicate that higher concentrations of membrane lipids are linked to poorer functioning and more severe symptoms. Analyses of relationship between membrane lipid levels at T_1_ and symptom levels at T_2_ indicate no significant associations.

## Discussion

The main findings of the present study were repeated higher levels of serum triglyceride in schizophrenia patients than in healthy controls during a 5 year period, while membrane lipid levels (PUFA) were lower in the acute stage of the disease (T_1_). There were no significant associations between lipid levels and symptoms in the acute stage (T_1_), while at the chronic stage (T_2_) both serum and membrane lipid levels were associated with symptoms and functioning. Higher serum lipid levels in the acute stage (T_1_) were also associated with more severe symptoms in the chronic stage (T_2_). These findings suggest serum lipid abnormalities as a putative core disease mechanism related to disease traits, while membrane lipids seem to fluctuate in different disease phases. This may be related to changes in neuroinflammatory and oxidative processes which are reported to contribute to disease progression and underlie symptom severity [34, 35].

We found higher levels of serum triglyceride in the patient group both at an acute stage (T_1_) and at follow-up 5 years later (T_2_). In longitudinal studies, dyslipidemia in patients with schizophrenia has primarily been studied as a side effects of antipsychotic medication [36]. Some studies have shown that dyslipidemia and other metabolic risk factors may be present in early stages of the disease, before treatment is initiated [37, 38]. Antipsychotics have been shown to up-regulate the expression of cholesterol transport proteins [39]. Further, the degree of dyslipidemia may be predictive of the effect of treatment [40]. In the present study, a large proportion of patients did not receive antipsychotic treatment when lipids were measured (47 % at T_1_, 20 % at T_2_). Thus, the sustained higher levels of serum triglyceride, suggest that dyslipidemia may be associated with the disease itself, and not only a result of medication. Further, smoking, gender, antipsychotic medication, and dietary factors does not explain the levels of membrane lipids [9, 26]. The notion that triglyceride levels may be a disease trait is in accordance with recent findings of polygenic overlap between blood lipid levels and schizophrenia, suggesting similar molecular genetic factors [41].

Higher levels of serum lipids were associated with more severe psychiatric symptoms in the chronic phase of the disease. We are not aware of similar findings. Other studies have shown that an increase in serum lipids is related to a reduction of symptoms among patients during treatment with antipsychotic medication [28, 40]. Our findings may be due to more severely ill patients having a more unhealthy lifestyle or being treated with higher doses of antipsychotics, which both can entail higher serum lipid levels. Earlier we have shown that serum lipid levels were not significantly higher among the patients using antipsychotic medication at T_2_ [26]. The present association between serum lipids and symptoms and functioning became stronger as the disease progressed to a more stable phase. This may reflect increased oxidative stress during the acute psychotic state, disrupting normal relationship between serum lipids and psychiatric symptoms [42, 43]. Among patients, triglyceride levels, and to a lesser degree cholesterol levels, at T_1_ were associated with symptom levels at T_2_. Serum lipid levels seem to be related not only to present symptoms, weight and medication, but also to the disease itself. These findings may indicate that disturbed serum lipid levels is a disease trait. This also raises the possibility that serum lipids to some degree may be used as a biomarker in schizophrenia.

To the best of our knowledge, the current study has the longest follow-up period of membrane lipids (PUFA) in schizophrenia yet reported. While long-term PUFA levels in the healthy control group were stable, the levels changed in the patient group. However, PUFA levels increased only in the patients with low PUFA levels at T_1_, while patients with higher PUFA had stable values. The bimodal distribution of PUFA and LCPUFA reported earlier at T_1_ [9] was not found at T_2_. Earlier reports on the differences in PUFA levels have shown discrepancies. Several studies have shown lower levels among patients [9, 11] while others reported no such difference [37]. Meta-analyses have shown lower levels of LCPUFA in schizophrenia patients than in healthy controls [10]. Lower levels of membrane lipids have been shown both among drug-naïve patients and in patients treated with antipsychotic drugs [38]. Discrepancies between studies can reflect that PUFA levels were measured at different stages during the course of the disease. During episodes with higher symptom intensity, levels of membrane lipids may be influenced by neuroinflammation, oxidative stress and lipid peroxidation [44, 45]. Treatment with atypical antipsychotic drugs may have a normalizing effect on the phospholipid composition of cell membranes, especially for drug-naïve patients [38]. In the present study, the PUFA levels in the chronic phase of schizophrenia were not lower than in healthy controls, possibly connected to long-term effects of antipsychotic drugs on PUFA [4] or to the remission from an acute psychotic episode [9].

Levels of membrane lipids (PUFA) were associated to symptoms at the follow-up stage (T_2_) but not at the acute stage (T_1_). At follow-up, higher levels of PUFA, especially LCPUFA, were associated with higher symptom intensity and poorer functioning [26]. Our findings at T_1_ may reflect lipid peroxidation, to which LCPUFA, such as DHA (docosahexaenoic acid) and EPA (eicosapentaenoic acid), are especially prone, and may support a role of oxidative stress and free radicals in schizophrenia [46]. Studies of fibroblasts from patients with schizophrenia have shown decreased lysolipid levels and disrupted extracellular matrix when exposed to oxidative stress [47]. Antioxidant defenses are regulators of immunological pathways [16], Schizophrenia is among several neuropsychiatric disorders for which neuroinflammatory processes have been suggested to play a role [44, 48]. Markers of increased inflammation have been found in post-mortem studies [49], in neuroimaging studies [50], in cerebrospinal fluid [51] and peripherally in blood [52] from schizophrenia patients, and disease severity has also been associated with inflammatory markers [53]. The relationship between lipid metabolism and inflammation is established through a series of experimental [54] and clinical studies [55], including shared genetic risk [56]. There is a link between inflammation and both serum lipids [57] and membrane lipids [58]. Inflammation may affect lipid levels through effects on arachidonic acid (ARA) and other phospholipids [59, 60]. PUFA are not only important components of neuronal cell membranes, but also play an important role in regulation of inflammation through the formation of eicosanoids [61]. Inflammation and oxidative stress may play a role in disease progression through lipid peroxidation and cholesterol oxidation, leading to neuronal cell death [62, 63]. These mechanisms may change during the course of the disease. This may explain the conflicting findings regarding membrane lipids and symptoms in different stages of schizophrenia. The unstable character of PUFA may explain why membrane lipids at T_1_ did not predict symptoms at T_2_, in contrast to serum lipids. Taken together, this indicates that, in contrast to serum lipids, the levels of membrane lipids are associated with present psychotic symptoms, and not future or past symptom severity.

The current study has some limitations. T_1_ differ from T_2_, both in terms of duration of illness and acute versus chronic stage. Thus, the effect of duration of illness and the stage may both explain why levels and correlations differ between T_1_ and T_2_. The relative importance of each of the characteristics and the influence of confounding factors may not be established by the present design. The weight of the subjects was not obtained at T_2,_ and thus obesity as a factor connected to dyslipidemia cannot be adjusted for. However, it is unlikely that weight affects the associations with symptom levels. Our screening protocol ensured a very small likelihood that any of the participants had familial dyslipidemia. However, diet, dietary supplements other than fatty acids, and other lifestyle factors that may influence the lipid levels, and family history of dyslipidemia were not controlled for. Further, at T_1_ there were 16 different raters, as opposed to one single rater at T_2_. More raters will introduce statistical noise, weakening relationships between lipids and symptoms, which could explain lack of association at T_1._ The control group was not matched for smoking habits and other lifestyle factors, and not for socioeconomic factors such as education level. Though these parameters may influence the lipid levels, matching them against a group of schizophrenia patients would give a sample not representative of the general population. The relative importance of disease and medication cannot be clarified with the present design. The design of the study is not fit to make conclusions about causality. Since this is a naturalistic study, confounding factors not accounted for in the present analysis can affect the lipid levels and clinical symptoms, and the findings need to be replicated in independent studies.

## Conclusion

To conclude, serum lipid levels were elevated among patients both in the acute and chronic stage, while membrane lipids were low in the acute phase of schizophrenia. Serum lipid levels, both in the acute and the chronic stages of schizophrenia, correlated with chronic symptom levels, while membrane lipids showed a more mixed relationship to symptom levels. These findings suggest that higher serum lipids may be a disease trait of schizophrenia, and a possible biomarker, and provide new insight into the role of lipid profiles in schizophrenia pathophysiology.

## Abbreviations

ARA: Arachidonic acid
DHA: Docosahexaenoic acid
EPA: Eicosapentaenoic acid
GAF: Global assessment of functioning
LCPUFA: Long chain polyunsaturated fatty acids, omega-3 + omega-6, with 20 or more carbon atoms, in red blood cells
MINI: Mini international neuropsychiatric interview
PANSS: Positive and negative syndrome scale
PUFA: Polyunsaturated fatty acids, omega-3 +, omega-6 polyunsaturated fatty acids in red blood cells
RBC: Red blood cells
SCID: Structural clinical interview for DSM-IV
